# Regulation of cerebrovascular compliance compared with forearm vascular compliance in humans: a pharmacological study

**DOI:** 10.1152/ajpheart.00377.2022

**Published:** 2022-12-02

**Authors:** M. Erin Moir, Stephen A. Klassen, Mair Zamir, J. W. Hamner, Can Ozan Tan, J. Kevin Shoemaker

**Affiliations:** ^1^School of Kinesiology, University of Western Ontario, London, Ontario, Canada; ^2^Department of Kinesiology, Brock University, St. Catharines, Ontario, Canada; ^3^Department of Mathematics, University of Western Ontario, London, Ontario, Canada; ^4^Department of Medical Biophysics, University of Western Ontario, London, Ontario, Canada; ^5^Cerebrovascular Research Laboratory, Spaulding Hospital Cambridge, Cambridge, Massachusetts; ^6^RAM, Electrical Engineering, Mathematics, and Computer Science, University of Twente, Enschede, The Netherlands; ^7^Department of Physiology and Pharmacology, University of Western Ontario, London, Ontario, Canada

**Keywords:** glycopyrrolate, nicardipine, phentolamine, vascular compliance, vascular resistance

## Abstract

Increasing evidence indicates that cerebrovascular compliance contributes to the dynamic regulation of cerebral blood flow but the mechanisms regulating cerebrovascular compliance in humans are unknown. This retrospective study investigated the impact of neural, endothelial, and myogenic mechanisms on the regulation of vascular compliance in the cerebral vascular bed compared with the forearm vascular bed. An index of vascular compliance (*C*_i_) was assessed using a Windkessel model applied to blood pressure waveforms (finger photoplethysmography) and corresponding middle cerebral artery blood velocity or brachial artery blood velocity waveforms (Doppler ultrasound). Data were analyzed during a 5-min baseline period (10 waveforms) under control conditions and during distinct sympathetic blockade (*experiment 1*, phentolamine; 10 adults), cholinergic blockade (*experiment 2*, glycopyrrolate; 9 adults), and myogenic blockade (*experiment 3*, nicardipine; 14 adults). In e*xperiment 1*, phentolamine increased *C*_i_ similarly in the cerebral vascular bed (131 ± 135%) and forearm vascular bed (93 ± 75%; *P* = 0.45). In *experiment 2*, glycopyrrolate increased cerebrovascular *C*_i_ (72 ± 61%) and forearm vascular *C*_i_ (74 ± 64%) to a similar extent (*P* = 0.88). In *experiment 3*, nicardipine increased *C*_i_ but to a greater extent in the cerebral vascular bed (88 ± 88%) than forearm vascular bed (20 ± 45%; *P* = 0.01). Therefore, adrenergic, cholinergic, and myogenic mechanisms contribute to the regulation of cerebrovascular and forearm vascular compliance. However, myogenic mechanisms appear to exert more specific control over vascular compliance in the brain relative to the forearm.

**NEW & NOTEWORTHY** Vascular compliance represents an important determinant in the dynamics and regulation of blood flow through a vascular bed. However, the mechanisms that regulate vascular compliance remain poorly understood. This study examined the impact of neural, endothelial, and myogenic mechanisms on cerebrovascular compliance compared with forearm vascular compliance. Distinct pharmacological blockade of α-adrenergic, endothelial muscarinic, and myogenic inputs altered cerebrovascular and forearm vascular compliance. These results further our understanding of vascular control and blood flow regulation in the brain.

## INTRODUCTION

A combination of steady-state and pulsatile flow mechanics is involved in the control of blood flow through a vascular bed (1). The human cerebral circulation requires precise regulation of blood flow to ensure adequate perfusion and oxygen delivery while defending against microvasculature damage caused by unduly high blood pressure (BP). Historically, studies investigating cerebral blood flow regulation in humans have focused on modifications to vascular resistance, which affects the steady component of flow (1). The importance of considering vascular compliance in cerebrovascular adjustments was first recognized through the use of Windkessel models, incorporating both vascular resistance and compliance, which more accurately described cerebral blood velocity responses during BP alterations compared with single-resistance models (2) or BP alone (3). Using a modified Windkessel approach, our recent investigation demonstrated that increases in cerebrovascular compliance contributed to the preservation of systolic blood velocity during transient reductions in BP (4). However, the mechanisms governing cerebrovascular compliance and the pulsatile component of cerebral blood flow remain unstudied.

In contrast, the mechanisms governing vascular compliance in the peripheral circulation have been explored including neural and myogenic inputs. For example, sympathoexcitation by lower body negative pressure and a cold pressor test, reduced forearm vascular compliance while elevation of the arm above the heart, changing forearm perfusion pressure and eliciting myogenic responses, produced an increase in forearm vascular compliance (1). Furthermore, phentolamine infusion increased forearm vascular compliance when the arm was elevated above the heart but not when the arm was below the heart, suggesting a dominant impact of myogenic regulation over vascular compliance in the forearm vascular bed (5). These studies suggest that processes affecting vascular contractile state inversely affect vascular compliance.

Studies regarding vascular compliance in the forearm may not translate well to the brain because of the difference in baseline contractile state, neural innervation, and intracranial pressure. Specifically, relative to the forearm, the cerebral vascular bed is chronically dilated, the impact of adrenergic sympathetic neural innervation differs, and the rigid cranium produces a state of elevated extramural pressure for the brain’s circulation. These conditions are expected to influence the stiffness of the cerebral vascular bed relative to the peripheral vascular bed. Therefore, this study aimed to gain new insight to the mechanisms governing cerebrovascular compliance by comparing the mechanisms regulating vascular compliance in the cerebral and forearm vascular beds.

The present study used a pharmacological approach to study the impact of neural, endothelial, and myogenic mechanisms on cerebrovascular compliance. Specifically, we examined the impact of α-adrenergic receptors, endothelial muscarinic receptors, and vascular smooth muscle calcium (Ca^2+^) channels (involved in the myogenic response) on vascular compliance in healthy humans. We applied a Windkessel modeling approach to calculate cerebrovascular and forearm vascular compliance under control conditions and during distinct drug infusions of phentolamine (nonselective α-adrenergic receptor blockade), glycopyrrolate (endothelial muscarinic receptor blockade), and nicardipine (vascular smooth muscle Ca^2+^ channel blockade). On the basis that vascular contractile state can limit the expression of vascular compliance, the present study tested the hypothesis that sympathetic blockade would increase cerebrovascular compliance because of smooth muscle cell relaxation. On the basis that endothelial muscarinic receptors exert a vasodilatory effect, the present study tested the hypothesis that cholinergic blockade would reduce cerebrovascular compliance because of greater smooth muscle cell activation. On the basis that vascular smooth muscle Ca^2+^ channels are involved in myogenic responses; the present study tested the hypotheses that their blockade would increase cerebrovascular compliance through smooth muscle cell relaxation. Concurrent measures in the forearm assessed the systemic versus localized cerebral effects of these pharmacological agents.

## MATERIALS AND METHODS

The present study involves retrospective analysis of data derived from three experiments separately performed and previously reported (6–8).

### Ethical Approval

The studies were approved by the Institutional Review Boards of the Hebrew Rehabilitation Center for Aged and Spaulding Rehabilitation Hospital. The studies conformed to the standards of the Declaration of Helsinki, and participants provided written informed consent.

### Participants

*Experiment 1* was performed in 11 healthy adults aged 21–40 yr (4 females). *Experiment 2* was performed nine healthy adults aged 21–30 yr (5 females). *Experiment 3* was performed in 16 healthy adults aged 21–30 yr (7 females). Before all experiments, participants were asked to abstain from caffeine consumption for at least 12 h, alcohol consumption for at least 24 h, and physical exercise for at least 24 h.

### Experimental Protocol

As previously reported (6–8), participants were instrumented with an electrocardiogram (lead II; Dash 2000, GE Medical Systems, Waukesha, WI) to measure heart rate (HR), a finger photoplethysmograph (Portapres, Ohmeda, Finapres Medical Systems, Enschede, The Netherlands) to measure arterial blood pressure (BP), Doppler ultrasound (MultiDop T2, DWL Elektronische Systeme, Singen, Germany) to measure peak middle cerebral artery blood velocity (MCAv; 2 MHz probe) and mean brachial artery blood velocity (BAv; 4 MHz probe), and a nasal cannula to measure end-tidal carbon dioxide partial pressures ($\text{P}ET_{\text{C}{\text{O}}_{2}}$; infrared CO_2_ analyzer, Model 17515, VacuMed, Ventura, CA). All signals were collected and stored for offline analysis with data acquisition systems (Windaq, DATAQ Instruments, Akron, OH, for *experiment 1* and PowerLab, ADInstruments, Colorado Springs, CO, for *Experiments 2 and 3*). A 20-guage catheter was inserted into an antecubital vein for drug infusion.

The protocol involved 5 min of supine baseline under control conditions and following drug infusion. In *experiment 1*, a 0.14 μg/kg bolus followed by a 0.014 μg/kg/min infusion of phentolamine adequately blocks α-adrenergic effects on the vasculature and reduces total peripheral resistance (9). In *experiment 2*, stepwise infusions of 0.2 mg glycopyrrolate over 20–30 min to achieve a target HR > 100 beats/min, suggesting adequate cerebral endothelial muscarinic receptor blockade (10). In *experiment 3*, a 3-mg bolus infusion of nicardipine hydrochloride over 8–10 min to block l-type calcium channels on the vasculature. This represents an overall modest clinical dose for acute outcomes and similar doses of nicardipine reduced mean arterial pressure in humans (11, 12).

### Data Analysis

Analysis was completed on *n* = 10 participants (4 females) for *experiment 1*, *n* = 9 participants (5 females) for *experiment 2*, and *n* = 14 participants (6 females) for *experiment 3*. A total of three participants were excluded from analysis (*n* = 1 in *experiment 1, n* = 2 in *experiment 3*) as they did not meet signal quality required for the analysis. Analysis was completed on a selection of 10 cardiac cycles during steady-state conditions of both the control and drug infusion periods. The individual BP and corresponding MCAv and BAv waveforms were extracted in an alternating pattern (i.e., every second cardiac cycle) to capture 4 ± 1 respiratory cycles. Previously, we have shown strong reproducibility between separate baseline selections using 10 cardiac cycles (4). To account for temporal delays between pressure pulse arrival at the brachial and middle cerebral arteries, the BP waveform was shifted to align with the foot of the corresponding MCAv waveform before extraction. Once extracted, the waveforms were input into a four element lumped parameter modified Windkessel model (custom software, previously described in detail) (13). For each beat extracted, the model calculated an index of cerebrovascular compliance (*C*_i_) and forearm vascular *C*_i_. Previously, using a similar analytical approach, we demonstrated strong reproducibility between baseline conditions separated by 5–10 min (4). In addition, an index of vascular resistance (*R*_i_) was calculated for the cerebral and forearm vascular beds as the quotient of mean BP over mean MCAv and BAv, respectively. For each beat extracted and input into the model, additional hemodynamic measures were analyzed, including HR, systolic BP (SBP), diastolic BP (DBP), pulse pressure (PP), systolic MCAv, diastolic MCAv, systolic BAv, and diastolic BAv. For each measure, averages were calculated across the 10 values at baseline. $\text{P}ET_{\text{C}{\text{O}}_{2}}$ was assessed for the selection of beats analyzed.

### Statistical Analysis

A two-way repeated-measures ANOVA evaluated the effect of drug infusions (control vs. drug) and vascular bed (cerebral vs. forearm) on *C*_i_ and *R*_i_. Post hoc paired *t* tests were performed to evaluate simple main effects of drug in each vascular bed. Paired *t* tests evaluated differences in the absolute and percent change in *C*_i_ between the cerebral and forearm vascular beds. The agreement of change in *C*_i_ with each drug between the cerebral vascular and forearm vascular beds was evaluated by Pearson’s product moment correlations or Spearman’s correlations where noted. Paired *t* tests evaluated the effect of drug infusions (control vs. drug) on hemodynamic variables and $\text{P}ET_{\text{C}{\text{O}}_{2}}$. Outliers were identified using the ROUT method. Outliers did not affect statistical results and therefore were included in statistical analysis. Statistical analyses were performed using SPSS Statistics 25 (SPSS, Chicago, IL) and GraphPad Prism 9 (GraphPad Software, San Diego, CA). Effect sizes were calculated with G*Power 3.0.10 (14). Statistical significance was defined as *P* < 0.05, and data are presented as means (SD).

## RESULTS

**Figure 1.**
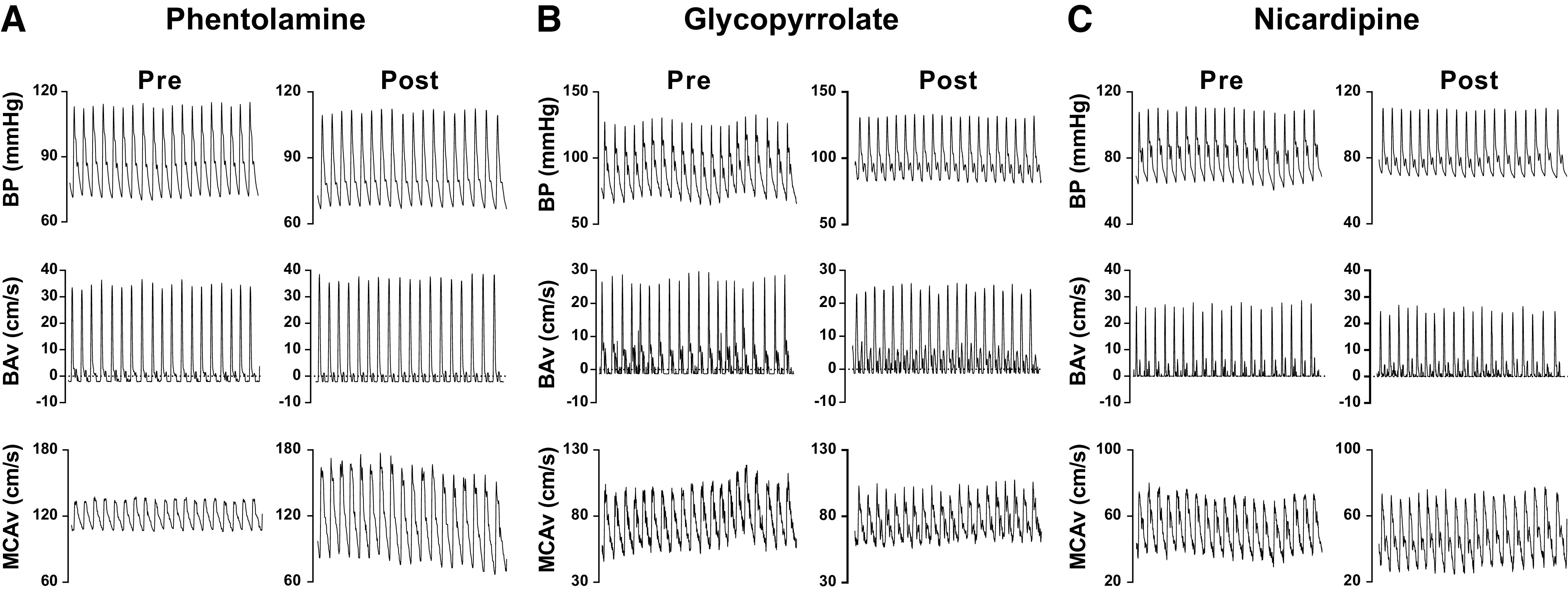
Representative blood pressure (BP), brachial artery blood velocity (BAv), and middle cerebral artery blood velocity (MCAv) waveforms from one individual in *experiment 1* (*A*; phentolamine infusion), *experiment 2* (*B*; glycopyrrolate infusion), and *experiment 3* (*C*; nicardipine infusion).

Figure 1 displays representative systemic BP, BAv, and MCAv before and during drug infusions in *experiment 1* (Fig. 1*A*; phentolamine), *experiment 2* (Fig. 1*B*; glycopyrrolate), and *experiment 3* (Fig. 1*C*; nicardipine).

### The Effect of Vascular Bed on Compliance and Resistance during Control Conditions

**Figure 2.**
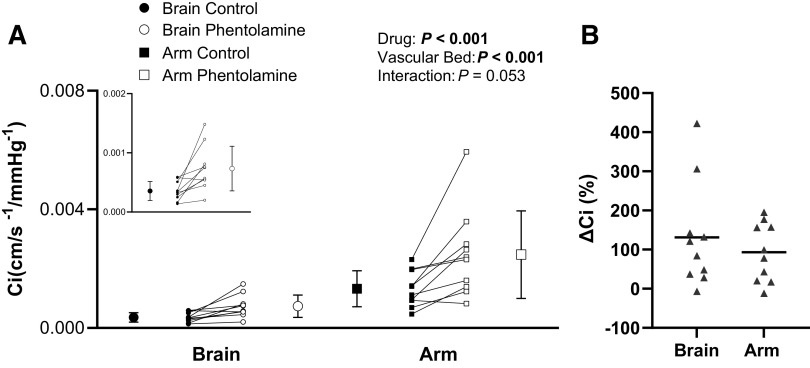
*A*: forearm vascular compliance (*C*_i_) and cerebrovascular *C*_i_ before (control) and after phentolamine infusion (phentolamine) under baseline conditions. *Inset*: cerebrovascular *C*_i_ shown on a smaller scale. Means (SD) (error bars) and individual data (solid lines) are presented. A two-way repeated-measures ANOVA examined the effect of drug (control vs. phentolamine) and vascular bed (cerebral vs. forearm) on *C*_i_ (*n* = 10, 4 females). *B*: percent increase in forearm vascular *C*_i_ and cerebrovascular *C*_i_ with phentolamine. Means (solid line) and individual data are shown. A paired *t* test evaluated differences between the cerebral and forearm vascular beds in the increase of *C*_i_ with phentolamine (*n* = 10, 4 females).

**Figure 3.**
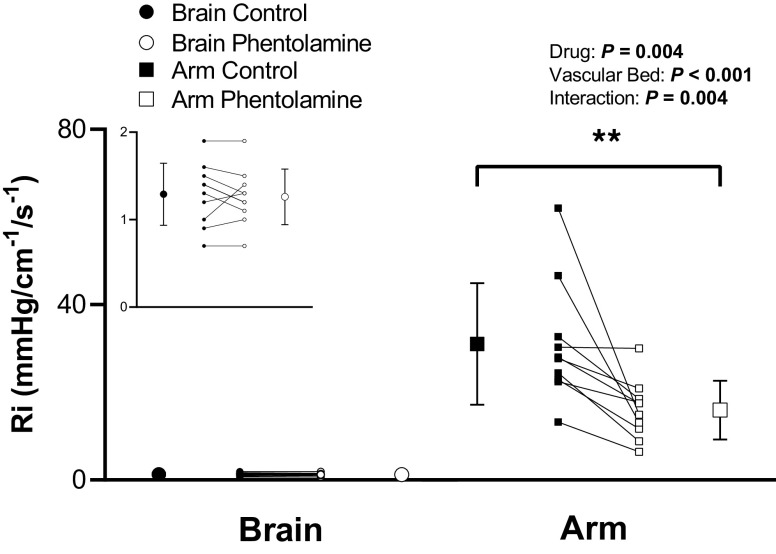
Vascular resistance (*R*_i_) in the cerebral vascular bed and forearm vascular bed before (control) and after phentolamine infusion (phentolamine) under baseline conditions. *Inset*: cerebrovascular *R*_i_ shown on a smaller scale. Means (SD) (error bars) and individual data (solid lines) are presented. A two-way repeated-measures ANOVA examined the effect of drug (control vs. phentolamine) and vascular bed (cerebral vs. forearm) on *R*_i_ (*n* = 10, 4 females). **P < 0.01.

**Figure 4.**
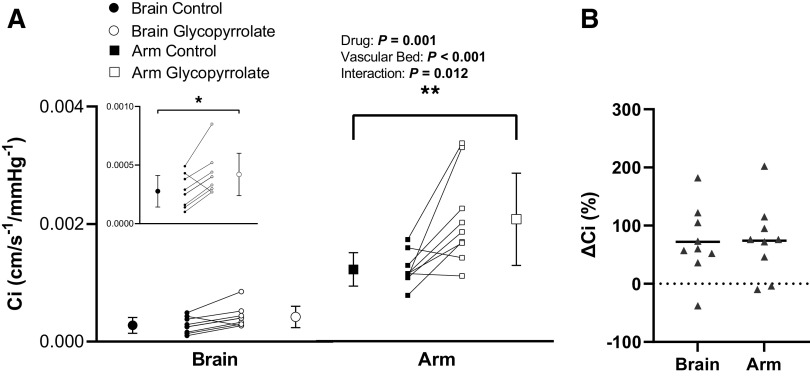
*A*: forearm vascular compliance (*C*_i_) and cerebrovascular *C*_i_ before (control) and after glycopyrrolate infusion (glycopyrrolate) under baseline conditions. *Inset*: cerebrovascular *C*_i_ shown on a smaller scale. Means (SD) (error bars) and individual data (solid lines) are presented. A two-way repeated-measures ANOVA examined the effect of drug (control vs. glycopyrrolate) and vascular bed (cerebral vs. forearm) on *C*_i_ (*n* = 9, 5 females). *B*: percent increase in forearm vascular *C*_i_ and cerebrovascular *C*_i_ with glycopyrrolate. Means (solid line) and individual data are shown. A paired *t* test evaluated differences between the cerebral and forearm vascular beds in the increase of *C*_i_ with glycopyrrolate (*n* = 9, 5 females). *P < 0.05; **P < 0.01.

**Figure 5.**
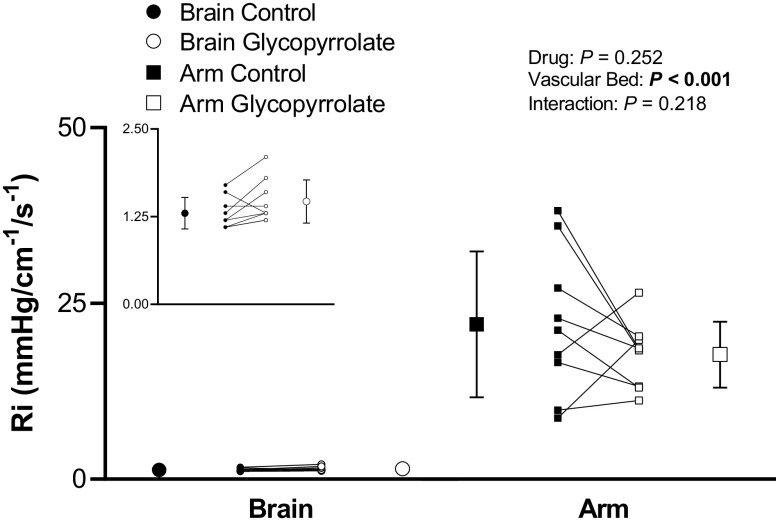
Vascular resistance (*R*_i_) in the cerebral vascular bed and forearm vascular bed before (control) and after glycopyrrolate infusion (glycopyrrolate) under baseline conditions. *Inset*: cerebrovascular *R*_i_ shown on a smaller scale. Means (SD) (error bars) and individual data (solid lines) are presented. A two-way repeated-measures ANOVA examined the effect of drug (control vs. glycopyrrolate) and vascular bed (cerebral vs. forearm) on *R*_i_ (*n* = 9, 5 females).

**Figure 6.**
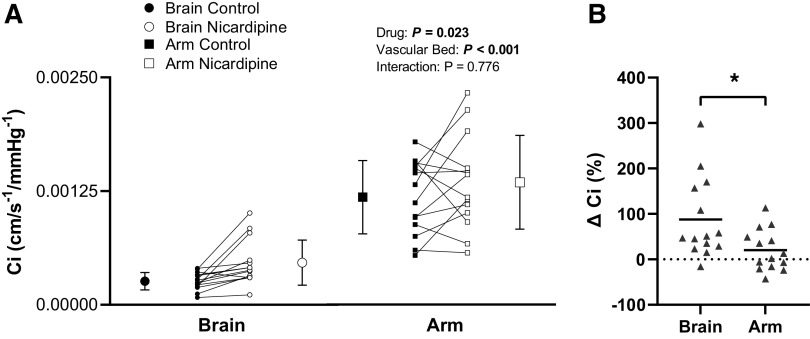
*A*: forearm vascular compliance (*C*_i_) and cerebrovascular *C*_i_ before (control) and after nicardipine infusion (nicardipine) under baseline conditions. Means (SD) (error bars) and individual data (solid lines) are presented. A two-way repeated-measures ANOVA examined the effect of drug (control vs. nicardipine) and vascular bed (cerebral vs. forearm) on *C*_i_ (*n* = 14, 6 females). *B*: percent increase in forearm vascular *C*_i_ and cerebrovascular *C*_i_ with nicardipine. Means (solid line) and individual data are shown. A paired *t* test evaluated differences between the cerebral and forearm vascular beds in the increase of *C*_i_ with nicardipine (*n* = 14, 6 females). *P < 0.05.

**Figure 7.**
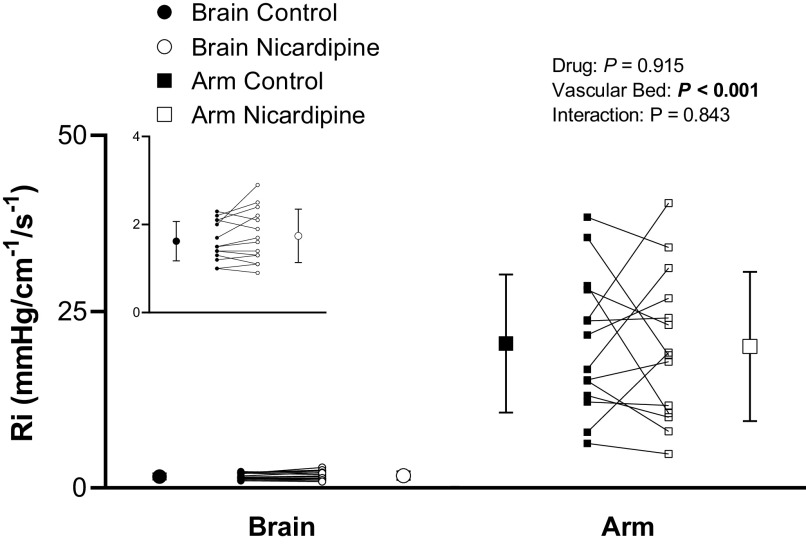
Vascular resistance (*R*_i_) in the cerebral vascular bed and forearm vascular bed before (control) and after nicardipine infusion (nicardipine) under baseline conditions. *Inset*: cerebrovascular *R*_i_ shown on a smaller scale. Means (SD) (error bars) and individual data (solid lines) are presented. A two-way repeated-measures ANOVA examined the effect of drug (control vs. nicardipine) and vascular bed (cerebral vs. forearm) on *R*_i_ (*n* = 14, 6 females).

When considering data from the control conditions (i.e., before drug infusion) in *experiments 1*, *2*, and *3*, there was a significant effect of vascular bed on vascular *C*_i_ and *R*_i_. Forearm vascular *C*_i_ was four to five times greater than cerebrovascular *C*_i_ (vascular bed, *P* < 0.001 in all experiments; Figs. 2*A*, 4*A*, and 6*A*) and forearm vascular *R*_i_ was 13–24 times greater than cerebrovascular *R*_i_ (vascular bed, *P* < 0.001 in all experiments; Figs. 3, 5, and 7).

### *Experiment 1*: *Sympathetic Blockade with Phentolamine*

Phentolamine infusion increased *C*_i_ in both the cerebral and forearm sites (drug, *P* < 0.001; vascular bed, *P* < 0.001; and drug-by-vascular bed interaction, *P* = 0.053; Fig. 2*A*). When compared with the control condition, phentolamine increased *C*_i_ in the cerebral vascular bed (*P*_post hoc_ *=* 0.01, *d* = 1.1) and forearm vascular bed (*P*_post hoc_ *=* 0.01, *d* = 1.0). The absolute increase in *C*_i_ during phentolamine infusion was not different between the two vascular beds (cerebral vascular bed, Δ 3.8e^−4^ ± 3.6e^−4^ cm/s/mmHg; and forearm vascular bed, Δ 11.5e^−4^ ± 11.2e^−4^ cm/s/mmHg; *P* = 0.08, *d = 0.8*). Phentolamine infusion increased *C*_i_ by 131 ± 135% in the cerebral vascular bed and 93 ± 75% in the forearm vascular bed (*P* = 0.45; Fig. 2*B*). The increase in cerebrovascular *C*_i_ during phentolamine infusion was not related to the increase in forearm vascular *C*_i_ when assessed with absolute change (Pearson’s correlation, *P* = 0.62) or percent change (Pearson’s correlation, *P* = 0.98).

When compared with the control condition, phentolamine differentially affected *R*_i_ by vascular bed (drug, *P* = 0.004; vascular bed, *P* < 0.001; and drug-by-vascular bed interaction, *P* = 0.004; Fig. 3). When compared with the control condition, forearm vascular *R*_i_ was reduced during phentolamine infusion (*P*_post hoc_ = 0.01, *d* = 1.0) whereas cerebrovascular *R*_i_ remained unchanged (*P*_post hoc_ = 0.64, *d* = 0.1). Phentolamine infusion did not affect mean BP (*P* = 0.20; Table 1) or PP (*P* = 0.51; Table 1), yet a large increase in HR was observed (Table 1). Phentolamine infusion did not impact $\text{P}ET_{\text{C}{\text{O}}_{2}}$ (control, 37 ± 4 mmHg; and phentolamine, 35 ± 5 mmHg; *P* = 0.40).

**Table 1.** Hemodynamic variables under control conditions and during drug infusion in all three experiments

	Control	Drug Infusion	*P* Value
*Experiment 1 - phentolamine*			
MAP, mmHg	94 (9)	90 (11)	0.20
SBP, mmHg	134 (18)	129 (22)	0.07
DBP, mmHg	77 (8)	74 (10)	0.34
PP, mmHg	57 (12)	55 (18)	0.51
HR, beats/min	60 (10)	75 (12)	**<0.001**
Mean MCAv, cm/s	79 (21)	75 (17)	0.28
Mean BAv, cm/s	4 (2)	7 (3)	**0.003**
*Experiment 2 - glycopyrrolate*			
MAP, mmHg	85 (6)	96 (11)	**0.03**
SBP, mmHg	124 (9)	127 (11)	0.57
DBP, mmHg	68 (6)	82 (11)	**0.01**
PP, mmHg	56 (9)	45 (5)	**0.01**
HR, beats/min	66 (10)	103 (7)	**<0.001**
Mean MCAv, cm/s	66 (7)	66 (8)	0.68
Mean BAv, cm/s	5 (2)	6 (2)	0.25
*Experiment 3 - nicardipine*			
MAP, mmHg	82 (11)	81 (12)	0.69
SBP, mmHg	116 (14)	115 (14)	0.84
DBP, mmHg	67 (10)	66 (11)	0.75
PP, mmHg	49 (9)	49 (10)	>0.99
HR, beats/min	58 (8)	70 (11)	**<0.001**
Mean MCAv, cm/s	54 (13)	51 (15)	**0.01**
Mean BAv, cm/s	5 (2)	5 (3)	0.78

Values are means (SD); *n*, number of participants: *n* = 10 (4 females) in *experiment 1*, *n* = 9 (5 females) in *experiment 2*, and *n* = 14 (6 females) in *experiment 3*. BAv, brachial artery blood velocity; DBP, diastolic blood pressure; HR, heart rate; MAP, mean arterial pressure; MCAv, middle cerebral artery blood velocity; PP, pulse pressure; SBP, systolic blood pressure. Boldface indicates significance.

### *Experiment 2*: *Cholinergic Blockade with Glycopyrrolate*

When compared with the control condition, glycopyrrolate increased *C*_i_ though its affect differed by vascular bed (drug, *P* = 0.001; vascular bed, *P* < 0.001; and drug-by-vascular bed interaction, *P* = 0.01; Fig. 4*A*). Glycopyrrolate increased cerebrovascular *C*_i_ (*P*_post hoc_ *=* 0.04, *d* = 1.1) and forearm vascular *C*_i_ (*P*_post hoc_ *=* 0.01, *d* = 1.2) but the absolute change in *C*_i_ between control conditions and glycopyrrolate infusion was greater in the forearm vasculature compared with the cerebral vasculature (Δ 8.5e^−4^ ± 7.4e^−4^ vs. Δ 1.4e^−4^ ± 1.3e^−4^ cm/s/mmHg; *P* = 0.01). However, given the difference in control *C*_i_ between the cerebral vascular bed (2.8e^−4^ ± 1.4e^−4^ cm/s/mmHg) and the forearm vascular bed (12.2e^−4^ ± 2.8e^−4^ cm/s/mmHg; *P* < 0.001), the percent change was not different between the vascular beds (brain, 72 ± 61%; and arm, 74 ± 64%; *P* = 0.88; Fig. 4*B*). Furthermore, the absolute change in cerebrovascular *C*_i_ was positively associated with the absolute change in forearm vascular *C*_i_ (Spearman’s correlation, *P* = 0.02, *r* = 0.77). A similar relationship was observed with the percent change in *C*_i_ between the cerebral and forearm vascular beds (Pearson’s correlation, *P* = 0.01, *r* = 0.78).

Glycopyrrolate infusion did not affect cerebrovascular or forearm vascular *R*_i_ (drug, *P* = 0.25; vascular bed, *P* < 0.001; and drug-by-vascular bed interaction, *P* = 0.22; Fig. 5). During glycopyrrolate infusion, mean BP was increased (*P* = 0.03; Table 1) whereas PP was decreased (*P* = 0.01; Table 1). A significant relationship was observed between the change in PP and the change in *C*_i_ (brain, *P* = 0.003, *r* = −0.86; and arm, *P* = 0.02, *r* = −0.77). A marked increase in HR was also observed following glycopyrrolate infusion (Table 1). Notably, glycopyrrolate did not affect $\text{P}ET_{\text{C}{\text{O}}_{2}}$ (control, 38 ± 5 mmHg; and glycopyrrolate, 37 ± 7 mmHg; *P* = 0.09).

### *Experiment 3*: *Myogenic Blockade with Nicardipine*

When compared with the control condition, nicardipine infusion increased *C*_i_ in both vascular beds (drug, *P* = 0.02; vascular bed, *P* < 0.001; and drug-by-vascular bed interaction, *P* = 0.78; Fig. 6*A*). The absolute change in *C*_i_ between control conditions and nicardipine infusion was not different between the cerebral vascular bed (Δ2.1e^−4^ ± 2.2e^−4^ cm/s/mmHg) and forearm vascular bed (Δ1.6e^−4^ ± 4.8e^−4^ cm/s/mmHg; *P* = 0.76). However, the percent increase in *C*_i_ with nicardipine was greater in the cerebral vascular bed (88 ± 88%) compared with the forearm vascular bed (20 ± 45%; *P* = 0.01; Fig. 6*B*), and there was no relationship between the percent increase in the two vascular beds (Pearson’s correlation, *P* = 0.35). This was also true when evaluating the relationship between the absolute change in cerebrovascular *C*_i_ and the absolute change in forearm vascular *C*_i_ (Pearson’s correlation, *P* = 0.89). In addition, as further support for differential effects of nicardipine on cerebrovascular *C*_i_ and forearm vascular *C*_i_, post hoc analysis of simple main effects revealed a significant increase in cerebrovascular *C*_i_ (*P*_post hoc_ *=* 0.002) but no change in forearm vascular *C*_i_ (*P*_post hoc_ = 0.23) with nicardipine.

Nicardipine infusion did not alter cerebrovascular or forearm vascular *R*_i_ (drug, *P* = 0.92; vascular bed, *P* < 0.001; and drug-by-vascular bed interaction, *P* = 0.84; Fig. 7). Mean BP and PP were not altered with nicardipine infusion (mean BP, *P* = 0.69; and PP, *P* > 0.99; Table 1), but an increase in HR was observed (Table 1). $\text{P}E{\text{T}}_{\text{C}{\text{O}}_{2}}$ was not impacted by nicardipine infusion (control, 36 ± 4 mmHg; and nicardipine, 35 ± 4 mmHg; *P* = 0.33).

## DISCUSSION

There are four major findings of the present study. First, across all three experiments, *C*_i_ was four- to fivefold greater in the forearm vascular bed than the cerebral vascular bed during control conditions. Second, nonselective α-adrenergic blockade with phentolamine increased *C*_i_ similarly in the cerebral and forearm vascular beds. Third, endothelial muscarinic receptor blockade with glycopyrrolate produced similar effects on cerebrovascular *C*_i_ and forearm vascular *C*_i_. Fourth, blockade of l-type Ca^2+^ channels with nicardipine induced a significant increase in cerebrovascular *C*_i_ but no difference in forearm vascular *C*_i_. Therefore, these data suggest that cerebrovascular *C*_i_ is affected by α-adrenergic, endothelial, and myogenic mechanisms with regionally specific differences of l-type Ca^2+^ channels.

### Factors Affecting Vascular Compliance

A few key factors should be considered when interpreting the findings from this study. The load-bearing function of elastin is greater at low pressures or unstretched vessels, producing highly compliant conditions, whereas collagen exerts proportionately greater support of wall tension with progressive distension leading to a curvilinear relationship between pressure and diameter (15–17). Also, the active contractile element of smooth muscle cells affects wall tension regardless of pressure and vessel diameter (17, 18). For example, sympathoexcitation reduced vascular compliance (1, 5, 19–21) whereas sympathoinhibitory conditions of brachial plexus blockade or radial artery denervation increased vascular compliance (22–24) in human peripheral conduit arteries. However, the original state of vessel dilation may affect the impact of modifying the contractile element on vascular compliance. In this scenario, the low compliance of a dilated vascular segment, due to collagen supporting wall tension, may be reversed by actively constricting this segment and reducing circumferential wall length so that elastin may support wall tension (18).

### Effect of Vascular Bed under Control Conditions

In all three experiments, performed in the supine posture, forearm vascular *C*_i_ was four- to fivefold greater than cerebral vascular *C*_i_ during control conditions. This may be expected based on several factors. For example, compared with systemic arteries, the cerebral arteries lack an external elastic lamina and exhibit fewer elastic fibers in the tunica media (25). Also, intracranial pressure (ICP) uniquely affects cerebrovascular *C*_i_ whereby the pressurized cranium restricts the cerebral vessels from expressing their elasticity in the supine posture (13). Furthermore, compared with the forearm vascular bed, the cerebral arteries exist in a highly dilated state at baseline where collagen may predominate over elastin in supporting wall tension (18). As expected (26), given the differences in vascular contractile state at baseline, *R*_i_ was greater in the forearm than the cerebral vascular bed.

### Sympathetic Blockade with Phentolamine

The present finding of increased *C*_i_ following phentolamine infusion supports the hypothesis that α-adrenergic mechanisms regulate human vascular compliance. Phentolamine increased *C*_i_ to a similar extent in the cerebral and forearm vascular beds. Since activation of smooth muscle cells reduces vascular *C*_i_ at a given diameter (1, 5, 19–21), we speculate that the augmented *C*_i_ with phentolamine relates to relaxation of smooth muscle cells following α-adrenergic receptor blockade. This observation aligns with previous reports of increased brachial or radial vascular compliance following brachial plexus blockade or radial artery denervation in humans (22–24). We anticipate that the large cerebral arteries contributed importantly to this outcome given the relatively larger sympathetic innervation of the intracranial arteries than parenchymal segments (27).

Importantly, phentolamine increased *C*_i_ similarly in the cerebral and forearm vascular beds while exerting differential effects on *R*_i_. Cerebrovascular *R*_i_ was unaltered following phentolamine infusion, supporting earlier observations in pigs (28, 29). The present finding of unaltered cerebrovascular *R*_i_ and increased cerebrovascular *C*_i_ aligns with previous reports where brachial plexus blockade or radial artery denervation did not alter vessel diameter yet increased vascular compliance (22–24). In contrast to the cerebral vascular bed, phentolamine imposed large reductions in forearm vascular *R*_i_ in the present study in line with earlier reports (9). The finding of reduced forearm vascular *R*_i_ and increased forearm vascular *C*_i_ aligns with a previous report where nitroglycerin administration concomitantly increased brachial artery cross-sectional area and compliance (21). Importantly, these data suggest that sympathetic inputs differentially affect vascular mechanics (compliance vs. resistance) between the two vascular beds.

Passive pressure-dependent mechanisms, such as changes to intra-arterial or extravascular pressure must be considered to explain the current observations. However, phentolamine augmented *C*_i_ in both vascular beds independent of changes in mean BP and PP. Also, specific to the brain, earlier studies suggest that phentolamine infusion in healthy adults did not affect ICP (30). Therefore, the current changes in *C*_i_ are not explained by changes in the transmural pressure gradient.

### Cholinergic Blockade with Glycopyrrolate

The present finding of augmented *C*_i_ following glycopyrrolate supports a role for endothelial muscarinic mechanisms in the regulation of vascular compliance. Although glycopyrrolate imposed a larger absolute increase in forearm vascular *C*_i_ than cerebral vascular *C*_i_, this effect was not present when examining changes relative to control *C*_i_. Also, the change in *C*_i_, whether absolute or relative, was positively related between the two vascular beds providing further support that glycopyrrolate imposed similar effects on cerebrovascular and forearm vascular *C*_i_. The present observation contrasts with the hypothesis that glycopyrrolate would reduce *C*_i_. This hypothesis was formed on the basis that endothelial muscarinic receptors elicit vasodilation through smooth muscle relaxation and by blocking these receptors, glycopyrrolate would produce smooth muscle activation, reducing vascular compliance at a given diameter.

The mechanisms mediating this outcome are not clear. The changes in vascular *C*_i_ with glycopyrrolate were independent of any changes in vascular *R*_i_. Furthermore, glycopyrrolate does not cross the blood-brain barrier (31) suggesting the outcomes related to glycopyrrolate’s blockade of muscarinic receptors are independent of effects on the central nervous system. Furthermore, through its effects on muscarinic receptor subtypes M_1_ to M_5_ (32), glycopyrrolate can affect the action of acetylcholine on the heart (M_2_), in addition to endothelial muscarinic receptors (M_3_). However, the rise in heart rate would be expected to reduce *C*_i_ (33, 34), not the increase observed here.

However, pressure-related mechanisms may have contributed to the increased *C*_i_ during glycopyrrolate infusion. When compared with control conditions, glycopyrrolate infusion increased mean BP, as previously observed (10, 35), due largely to elevated diastolic BP that, in turn, produced a reduction in PP. Based on the curvilinear pressure-diameter relationship, it can be assumed that glycopyrrolate caused a compression of the pressure-diameter curve to higher diastolic but similar systolic pressure. In this scenario, the calculated *C*_i_ could be affected by the compressed oscillatory pressure and the steeper rise in pressure and flow at the onset of systole, but confirmation of this speculation requires additional study.

### Myogenic Blockade with Nicardipine

The increase in *C*_i_ during nicardipine infusion supports the hypothesis that a mechanism related to l-type Ca^2+^ channels regulate human vascular compliance. Although nicardipine produced similar absolute increases in *C*_i_ between the cerebral and forearm vascular beds, the percent increase in *C*_i_ was larger in the cerebral vascular bed than the forearm vascular bed. Also, the increase in *C*_i_ was not related between the two vascular beds whether assessed as the absolute or percent increase. Therefore, the greater increase in cerebrovascular *C*_i_ compared with forearm vascular *C*_i_ suggests a greater sensitivity to l-type Ca^2+^ channels (myogenic mechanisms) in the brain’s circulation.

Nicardipine infusion did not alter *R*_i_ in the present study. This observation was unexpected as nicardipine reduces systemic vascular resistance and BP in hypertensive populations (36–38). In addition, studies investigating the cerebral vascular bed have demonstrated vasodilation of the cerebral arteries following nicardipine infusion in patients with cerebral vasospasm (39) or in healthy older men (40). However, studies in younger, healthy adults report increased plasma norepinephrine (41, 42) and vasoconstriction of the cerebral vascular bed (43) following nicardipine infusion. Thus, although speculative, the hemodynamic observations herein may be due to modest dose of nicardipine in the present study (3 mg infused over 8–10 min) and concurrent reflexive autonomic adjustments to defend blood pressure. Nonetheless, it is possible that nicardipine, through its mechanism to relax smooth muscle cells, imposed an increase in vascular *C*_i_ without any changes to vascular *R*_i_ as has been demonstrated in previous studies where smooth muscle relaxation did not affect vessel diameter but did increase vascular compliance (22–24).

Pressure-related mechanisms, such as changes to intra-arterial or extravascular pressure, are not expected to have contributed to the increase in *C*_i_ with nicardipine infusion as mean BP and PP were not altered. Previous literature regarding ICP during nicardipine administration have produced conflicting results with some studies showing no change in ICP (40, 44, 45), and others showing an increase or decrease in ICP (39, 46). Although reductions in ICP may have contributed to the present results, given the highly variable ICP response to nicardipine, we suspect the effect likely would have been small. Despite these uncertainties, the current results point to a myogenic mechanism that operates with high sensitivity within the cerebrovascular bed to regulate *C*_i_.

### Methodological Considerations

First, all drug interventions increased HR which reduces vascular elasticity (33, 34). Therefore, in the current observation of increased *C*_i_ despite concurrent tachycardia, the impact of α-adrenergic, endothelial muscarinic and Ca^2+^ channel blockades on *C*_i_ may be underestimated. Second, because the pharmacological agents were infused systemically, we cannot eliminate the contribution of integrative physiological mechanisms to changes in *C*_i_. For example, baroreflex-mediated vasoconstrictor adjustments may have influenced our results. However, this effect would be expected to reduce *C*_i_. Therefore, although the studies remain to be performed, we expect that the reflexive responses to phentolamine and nicardipine led to a possible underestimation of *C*_i_. Third, measures of vessel cross-sectional area were not available in the current analysis. Therefore, BV waveforms, collected with Doppler ultrasound, were used in the Windkessel model in place of blood flow waveforms resulting in scaled values of compliance. This is justified because the model uses waveform harmonics and not the absolute value of blood flow. Fourth, brachial artery BP waveforms were used in the absence of access to MCA BP measures. This is reasonable given modeled outcomes of similar waveforms and absolute blood pressures in both vessels (47). Finally, the Windkessel model is self-validating in that the BV waveform derived by the model is matched to the measured waveform.

### Conclusions

This study provides novel insight into neural, endothelial, and myogenic mechanisms that regulate vascular compliance in both the cerebral and forearm vascular bed. The present study also highlights diverging neural (α-adrenergic) regulation of vascular resistance between the forearm circulation and cerebral circulation and also differing myogenic regulation of vascular compliance between the two sites. The observation of augmented *C*_i_ during α-adrenergic, endothelial muscarinic, and myogenic blockade broadens our understanding of the control of blood flow through a vascular bed, particularly, the cerebral vascular bed where precise regulation of blood flow is critical.

## DATA AVAILABILITY

Data are available on request.

## GRANTS

This work was supported by National Heart, Lung, and Blood Institute Grant HL-093113; Natural Sciences and Engineering Research Council of Canada (NSERC) Discovery Grant RGPIN-2018-06255; and Canadian Institutes of Health Research (CIHR) Grant 201503MOP-342412-MOV-CEEA (to J.K.S.). M.E.M. was supported by an Ontario Graduate Doctoral Scholarship (O.G.S.). S.A.K. is supported by NSERC Discovery Grants RGPIN-2022-05293 and DGECR-2022-00320.

## DISCLOSURES

No conflicts of interest, financial or otherwise, are declared by the authors.

## AUTHOR CONTRIBUTIONS

M.E.M., J.W.H., C.O.T., and J.K.S. conceived and designed research; J.W.H. and C.O.T. performed experiments; M.E.M. and S.A.K. analyzed data; M.E.M., S.A.K., M.Z., J.W.H., C.O.T., and J.K.S. interpreted results of experiments; M.E.M. prepared figures; M.E.M., S.A.K., M.Z., J.W.H., C.O.T., and J.K.S. drafted manuscript; M.E.M., S.A.K., M.Z., J.W.H., C.O.T., and J.K.S. edited and revised manuscript; M.E.M., S.A.K., M.Z., J.W.H., C.O.T., and J.K.S. approved final version of manuscript.
